# Editorial: Exercise and transplant sport—the journey to a more active life

**DOI:** 10.3389/fspor.2025.1564320

**Published:** 2025-02-24

**Authors:** Mike J. Price, Gareth Wiltshire, Roseanne E. Billany, Tania Janaudis-Ferreira

**Affiliations:** ^1^ Physical Activity Sport and Exercise Sciences, Coventry University, Coventry, United Kingdom; ^2^ School of Sport, Exercise and Health Science, Loughborough University, Loughborough, United Kingdom; ^3^ Department of Cardiovascular Sciences, University of Leicester, Leicester, United Kingdom; ^4^ School of Physical and Occupational Therapy, McGill University, Montreal, QC, Canada; ^5^ Respiratory Epidemiology and Clinical Research Unit, Centre for Outcomes Research and Evaluation, Research Institute of the McGill University Health Centre, Montreal, QC, Canada

**Keywords:** solid-organ transplant, high intensity exercise, training load, athlete support, rehabilitation, wearable technology, educational resources

**Editorial on the Research Topic**
Exercise and transplant sport—the journey to a more active life

The goal of this Research Topic was to further our knowledge of exercise-related responses in organ recipients from rehabilitation to competitive sport. Indeed, the studies presented reflect our aim to consider organ recipients in the first 12 months post-transplantation (Vecchiato et al.) to those competing nationally and internationally (Hames et al.) and the support received at such events (Duncan et al.). Furthermore, the impact of high-intensity training was reviewed (Kayeye et al.) and the utility of web-based exercise and training resources (Da Silva et al.) and telehealth monitoring (Vecchiato et al.) was evaluated.

Vecchiato et al. reported on the use of telehealth tools for exercise monitoring over 12 months following kidney transplantation. For the first six months, patients underwent supervised tailored exercise sessions and were monitored monthly using video calls. Participants were then divided into a wearable group (Smartwatch, utilizing continuous objective monitoring) or continued with monthly video calls. Both groups demonstrated good overall adherence and improvements in lipid profile, blood pressure and cardiorespiratory fitness. However, the wearable device appeared to provide a greater impact on the perceived quality of life, likely related to the ability of wearables to provide accurate real-time feedback and personalized insights, facilitating enhanced motivation and engagement. Both the video call and wearable device interventions improved self-management and progress tracking and contributed to the level of adherence observed, although with different benefits and obstacles.

Kayeye et al. reviewed the physiological and health-related quality of life, clinical outcomes and safety considerations associated with high-intensity training interventions and strenuous sporting activities in organ recipients. High-intensity exercise was shown to safely enhance physiological outcomes while reducing coronary heart disease, emphasizing a helpful range of confounding factors for future research studies to consider. Few studies reported adverse events which, although encouraging, prompted a recommendation for clear *a priori* definitions to ensure that all events can be accurately acknowledged. Potential selection bias was also noted as all studies involved participants who were stable and otherwise healthy. Thus, translation of the findings to all organ recipients is not possible. More extreme accomplishments undertaken by organ recipients, namely ironman triathlons, marathon running, multi-day cycling events and mountain climbing were also presented, showcasing the potential for remarkable athletic achievements post-transplant. Despite these achievements the authors noted the caveat that such accomplishments were attained by individuals who were physically active, or already competing professionally before their transplant. The capabilities demonstrated by these participants therefore ought not to be expected of recipients who were inactive pre-transplant.

Continuing along the exercise training spectrum, Hames et al. presented the training load and practices of organ recipients competing in the British and World Transplant Games. The total training load [exercise duration (min) × Borg's 0–10 Rating of Perceived Exertion; arbitrary units [AU]] was ∼2,900 AU, but with considerable variation across competitors. Furthermore, athletes in the World Games demonstrated a greater training load than those competing predominantly in the British Games (3,000 vs. 1,400 AU, respectively). For comparison, the tailored training load for patients in their first 12 months post-transplant (Vecchiato et al.) was ∼1,690 AU. The wide range of training modalities and training loads among participants was likely due to multiple and diverse event participation reflecting the ethos of transplant sports, in addition to diverse physical fitness profiles and reasons for participation. Importantly, when considering exercise recommendations, the majority of competitors exercised more than twice a week and approximately half undertook some form of resistance training—but only one third did this at least twice a week. Notably, the majority of Games participants (71%) were active in sports prior to their transplant, suggesting that future research should consider strategies to overcome barriers and enhance facilitators to exercise in those who are inactive prior to transplant along with those with competitive goals.

Continuing the theme of competitive sport, Duncan et al. presented perspectives on providing a psychological sports performance well-being service at the British Transplant Games (Coventry, 2023), describing the background to the service, underlying models and philosophy and reflections from trainees and practitioners. Service provision included emotional support, psychological education on physical activity and lifestyle factors and psychological skills training. Key reflections included the atmosphere (“climate”) of the Games ranging from joy, hope and celebration of life to the complex interplay between gratitude and guilt, an aspect that is not generally experienced in other competitions. Given the health vulnerabilities of this population, the service placed well-being first and performance second, with priority given to listening to the athletes’ stories. The resultant emotional experiences of the group and the emotional labor of the Games placed considerable importance on peer supervision. Practical aspects of signposting, access to the service and finding a quiet space for consultations at a live event were highlighted. For trainees the service provided an opportunity to work with a broader client base than they might usually experience. However, this re-emphasizes the need for greater resources, for athletes, early career practitioners and those new to working with organ recipients, enabling volunteers to be fully prepared to support all involved.

The need for clear educational resources was addressed by Da Silva et al. who examined the online content and quality of the website and video resources relating to physical activity, with a focus on promoting engagement and adherence to activity post-transplant. Of the 49 resources included, the majority were of low quality, understandability and actionability. More comprehensive content scores were noted for resources from Foundations and advocacy websites compared to scientific organizations and news/media articles. Importantly, balanced and unbiased information was frequently observed, although only a minority of sources discussed safety and educational aspects, lacking additional resources for reference.

Overall, the studies included in this Research Topic have demonstrated diverse interdisciplinary work, utilizing collaborative partnerships across aspects of applied research and practice. As practitioners and researchers in this area, we are responsible not only for ensuring the integration of this research into practice but also for facilitating future, truly international collaborations. The ball is firmly in our court to enhance such developments along with high-quality resources for organ recipients and those professionals beginning their practice journey within this population.

